# WHO air quality database: relevance, history and future developments

**DOI:** 10.2471/BLT.23.290188

**Published:** 2023-10-24

**Authors:** Kerolyn Shairsingh, Giulia Ruggeri, Michal Krzyzanowski, Pierpaolo Mudu, Mazen Malkawi, Juan Castillo, Agnes Soares da Silva, Manjeet Saluja, Karla Cervantes Martínez, Josselyn Mothe, Sophie Gumy

**Affiliations:** a Department of Environment, Climate Change and Health, World Health Organization, 20 Avenue Appia, 1211 Geneva, Switzerland.; b Imperial College, London, England.; c World Health Organization, Regional Office for Europe, European Centre for Environment and Health, Bonn, Germany.; d World Health Organization, Regional Office for the Eastern Mediterranean, Cairo, Egypt.; e Pan American Health Organization, Washington, DC, United States of America.; f World Health Organization, India Country Office, New Delhi, India.

## Abstract

Air pollution is the second most important risk factor for noncommunicable diseases, but air quality monitoring is lacking in many low- and middle-income countries. The World Health Organization (WHO) recently released its 2022 updated air quality database status report. This report contains data from about 6743 human settlements, a sixfold increase from 1102 settlements in its first publication in 2011, which shows that air pollution is increasingly recognized as a health priority at global and national levels. However, progress varies across the world. More than 90% of the settlements in the database are in high- and middle-income countries and areas mainly in China, Europe, India and North America. The database is crucial for increasing awareness of air pollution, and for calculating global exposures and the corresponding burden of disease attributable to air pollution. This article describes the progress made and challenges in collecting air quality data. The database uses official data sources which can be difficult to access and assess, because air quality monitoring is done by different government bodies or uses varying monitoring methods. These air quality data can be used by the health sector to engage in discussions on monitoring air quality to protect public health, and facilitate multisectoral engagement of United Nations agencies to support countries to conform with the 2021 WHO air quality guidelines. Although air pollution levels in most countries are higher than those recommended in the guidelines, any action policy-makers take to reduce air pollution will help reduce the burden of air pollution on health.

## Résumé

Bien que la pollution de l'air représente le deuxième facteur de risque le plus important pour les maladies non transmissibles, de nombreux pays à revenu faible et intermédiaire ne mènent aucun contrôle de la qualité de l'air. L'Organisation mondiale de la Santé (OMS) a récemment publié l'édition 2022 du rapport de situation relatif à sa base de données sur la qualité de l'air. Ce rapport renferme des informations sur près de 6743 établissements humains, un chiffre six fois supérieur aux 1102 établissements humains figurant dans la première publication de 2011, ce qui montre que la pollution de l'air est davantage reconnue comme une priorité en matière de santé, tant à l'échelle nationale qu'internationale. Pourtant, les avancées ne sont pas les mêmes partout dans le monde. Plus de 90% des établissements mentionnés dans la base de données se trouvent dans des pays à revenu faible et intermédiaire, ainsi que dans des régions principalement situées en Chine, en Europe, en Inde et en Amérique du Nord. Cette base de données est essentielle pour mieux sensibiliser à la pollution de l'air, mais aussi pour calculer l'exposition mondiale et l'impact des maladies qui lui sont attribuables. Le présent article décrit les progrès réalisés et les défis qui subsistent dans la collecte d'informations liées à la qualité de l'air. La base de données utilise des sources officielles, qui peuvent être difficiles d'accès et compliquées à évaluer car le contrôle de la qualité de l'air est effectué par plusieurs organismes gouvernementaux ou emploie des méthodes différentes. Les informations ainsi récoltées peuvent être exploitées par le secteur de la santé pour entamer des discussions sur le contrôle de la qualité de l'air. Objectif: préserver la santé publique et favoriser la mobilisation multisectorielle d'agences des Nations Unies pour aider les pays à se conformer aux lignes directrices de l'OMS relatives à la qualité de l'air, qui datent de 2021. Même si, dans la plupart des pays, les niveaux de pollution de l'air dépassent les recommandations formulées dans ces lignes directrices, toute action entreprise par les responsables politiques pour les faire baisser contribuera à réduire l'impact qu'exerce cette pollution sur la santé.

## Resumen

La contaminación del aire es el segundo factor de riesgo más importante de las enfermedades no transmisibles, pero en muchos países de ingresos bajos y medios no se vigila la calidad del aire. La Organización Mundial de la Salud (OMS) publicó hace poco su informe actualizado de 2022 sobre el estado de la base de datos de calidad del aire. Este informe contiene datos de unos 6743 asentamientos humanos, es decir, seis veces más que los 1102 asentamientos de su primera publicación en 2011, lo que demuestra que la contaminación del aire se reconoce cada vez más como una prioridad sanitaria a nivel mundial y nacional. Sin embargo, los progresos varían en todo el mundo. Más del 90% de los asentamientos de la base de datos se encuentran en países y regiones de ingresos altos y medios, principalmente en China, Europa, India y Norteamérica. La base de datos es esencial para aumentar la concienciación sobre la contaminación del aire y para calcular las exposiciones globales y la correspondiente carga de morbilidad atribuible a la contaminación del aire. Este artículo describe los progresos realizados y los desafíos que plantea la recopilación de datos sobre la calidad del aire. La base de datos utiliza fuentes de datos oficiales a las que puede resultar difícil acceder y evaluar porque el control de la calidad del aire lo realizan diferentes organismos gubernamentales o utilizan métodos de control que varían. El sector sanitario puede utilizar estos datos sobre la calidad del aire para participar en debates sobre la vigilancia de la calidad del aire con el fin de proteger la salud pública y facilitar el compromiso multisectorial de los organismos de las Naciones Unidas para ayudar a los países a cumplir las directrices de la OMS 2021 sobre la calidad del aire. Aunque los niveles de contaminación del aire en la mayoría de los países son superiores a los recomendados en las directrices, cualquier medida que adopten los responsables de formular políticas para reducir la contaminación del aire contribuirá a reducir la carga de la contaminación del aire sobre la salud.

## ملخص

إن تلوث الهواء هو ثاني أهم عامل خطر للإصابة بالأمراض غير المعدية، إلا أن هناك افتقار لمراقبة جودة الهواء في العديد من الدول ذات الدخل المنخفض والدخل المتوسط. أصدرت منظمة الصحة العالمية (WHO) مؤخرًا تقريرها حول حالة قاعدة بيانات جودة الهواء المحدثة لعام 2022. يحتوي هذا التقرير على بيانات من حوالي 6743 تجمعًا بشريًا، أي بزيادة ستة أضعاف عن عدد الـ 1102 تجمعًا في منشوره الأول في عام 2011، مما يوضح أن تلوث الهواء يتم تقييمه بشكل متزايد كأولوية صحية على المستويين العالمي والوطني. ومع ذلك، فإن التقدم يتباين في جميع أنحاء العالم. إن أكثر من 90% من التجمعات المدرجة في قاعدة البيانات، تقع في الدول والمناطق ذات الدخل المرتفع والمتوسط، وبشكل رئيسي في الصين وأوروبا والهند وأمريكا الشمالية. تعتبر قاعدة البيانات ضرورية لزيادة الوعي بتلوث الهواء، ولحساب حالات التعرض العالمية، والعبء المرتبط بالأمراض التي تنجم عن تلوث الهواء. توضح هذه المقالة التقدم المحرز والتحديات في جمع بيانات جودة الهواء. تستعين قاعدة البيانات بمصادر البيانات الرسمية التي قد يكون من الصعب الوصول إليها وتقييمها، وذلك لأن مراقبة جودة الهواء تتم بواسطة هيئات حكومية مختلفة أو تعتمد على أساليب مراقبة متنوعة. يمكن استخدام بيانات جودة الهواء هذه بواسطة القطاع الصحي للمشاركة في مناقشات حول مراقبة جودة الهواء لحماية الصحة العامة، وتسهيل المشاركة متعددة القطاعات لوكالات الأمم المتحدة لدعم الدول على صعيد الامتثال للمبادئ التوجيهية لجودة الهواء التابعة لمنظمة الصحة العالمية لعام 2021. على الرغم من أن مستويات تلوث الهواء في معظم الدول أعلى من تلك الموصى بها في المبادئ التوجيهية، فإن أي إجراء يتخذه واضعو السياسات للحد من تلوث الهواء سيساعد في تقليل عبء تلوث الهواء على الصحة.

## 摘要

空气污染是导致非传染性疾病的第二大最重要风险因素，然而，许多低收入和中等收入国家没有空气质量监测。世界卫生组织 (WHO) 最近发布了 2022 年的空气质量数据状况报告。该报告包含约 6,743 个人类定居点的数据，定居点数量是 2011 年首次发布时包含的 1,102 个居住区的七倍，这表明空气污染已日益被认为是全球层面和国家层面的卫生重点事项。然而，世界各地的进展情况参差不齐。数据中 90% 以上的定居点位于中高收入国家和地区，主要分布在中国、欧洲、印度和北美。该数据对于提高人们对空气污染的认识和计算全球暴露量以及由空气污染所导致的相应疾病负担至关重要。本文介绍了在收集空气质量数据方面取得的进展和面临的挑战。该数据库使用的官方数据来源可能难以获取和评估，因为空气质量监测由不同的政府机构进行，或使用不同的监测方法。卫生部门可利用这些空气质量数据参与有关监测空气质量的讨论，以保护公众健康，并促进联合国各机构的多部门参与，从而支持各国遵守世卫组织 2021 年发布的空气质量指南。尽管大多数国家的空气污染水平都高于指南中建议的水平，但决策者为减少空气污染而采取的任何行动都将有助于减轻空气污染对健康造成的负担。

## Резюме

Загрязнение воздуха является вторым по значимости фактором риска развития неинфекционных заболеваний, однако во многих странах с низким и средним уровнем дохода отсутствует мониторинг качества воздуха. Недавно Всемирная организация здравоохранения (ВОЗ) опубликовала обновленный доклад о состоянии базы данных по качеству воздуха в 2022 году. Настоящий отчет содержит данные по 6743 населенным пунктам, что в шесть раз превышает данные по 1102 населенным пунктам в первой публикации в 2011 г., и свидетельствует о том, что загрязнение воздуха все чаще признается приоритетной задачей здравоохранения на глобальном и национальном уровнях. Однако прогресс в разных странах мира различается. Более 90% населенных пунктов, включенных в базу данных, находятся в странах и районах с высоким и средним уровнем дохода, в основном в Европе, Индии, Китае и Северной Америке. Эта база данных имеет решающее значение для повышения осведомленности о загрязнении воздуха, а также для расчета глобального воздействия и соответствующего бремени заболеваний, связанных с загрязнением воздуха. В данной статье рассказывается о достигнутом прогрессе и проблемах, связанных со сбором данных о качестве воздуха. В базе данных используются официальные источники данных, доступ к которым и их оценка могут быть затруднены, поскольку мониторинг качества воздуха осуществляется различными государственными органами или использует различные методы мониторинга. Полученные данные о качестве воздуха могут использоваться сектором здравоохранения для участия в обсуждении вопросов мониторинга качества воздуха в целях охраны общественного здравоохранения и содействия многосекторальному участию представительств ООН в оказании поддержки странам в соблюдении рекомендаций ВОЗ по качеству воздуха на 2021 год. Несмотря на то что уровень загрязнения воздуха в большинстве стран превышает рекомендуемый в руководстве, любые действия ответственных лиц, направленные на снижение уровня загрязнения воздуха, помогут снизить влияние загрязнения воздуха на здоровье человека.

## Introduction

Air pollution is a recognized, global health risk factor and is associated with close to 7 million deaths every year.^1^ The availability of air quality data is essential to assess the magnitude, distribution and trends of the effects of pollution on population health. These data are also important for modelling air quality, which in turn is critical for informing regional and global policies on air quality.

The World Health Organization (WHO) has played a key role in increasing awareness on the health risks of air pollution. WHO has been responsible for regular updates of the air quality guidelines since the 1980s,^2^^–^^4^ the production of scientific reports on debated aspects of air pollution,^5^ and the compilation and publication of routinely measured data in the WHO air quality database.^6^ WHO has also been active in the development and implementation of tools to estimate the burden of disease attributable to exposure to air pollution.^7^^–^^10^ The availability of data on measured air pollution levels based on ground monitors has increased substantially in the past few decades.^11^^–^^13^ Each public release of the early versions of the WHO air quality database, or of the guidelines, has attracted media attention, putting air pollution and its harmful effects in the spotlight.

This paper presents an overview of close to 50 years of WHO’s activities in gathering air quality data and their use in global assessments and efforts to reduce health risks caused by air pollution.

## Evolution of air quality data

Efforts to compile a database of urban air pollution levels covering the entire globe date back to the 1970s, with the start of the WHO urban air quality monitoring pilot project in 1973. Subsequently, the project evolved in a component of the global environmental monitoring system air programme, which since 1975, WHO and the United Nations have operated as a component of the global environmental monitoring system.^14^ The programme’s original objective was to strengthen the monitoring of urban air pollution to improve data comparability among different countries, and to assess air pollution levels and trends at a global level. While the global environmental monitoring system was eventually phased out in its original form, UNEP has recently re-branded it as global environmental monitoring system air, with a shift in its focus to move beyond data management systems to partnership development, awareness-raising, capacity development and knowledge exchange.^15^

Since 2011, WHO has been compiling and publishing ground measurements of air quality,^7^ specifically annual mean concentrations of particulate matter (PM) with a diameter ≤ 2.5 µm (PM_2.5_) and ≤ 10 µm (PM_10_), with the objective of deriving robust estimates of exposure necessary to assess the burden of disease caused by air pollution.^16^^–^^18^ After 2015, the database, also referred to as the WHO air quality database, became an essential part of WHO’s role of monitoring indicators 11.6.2 (air quality in cities) and 3.9.1 (mortality attributed to air pollution) of the sustainable development goals (SDGs).

**Fig. 1:**
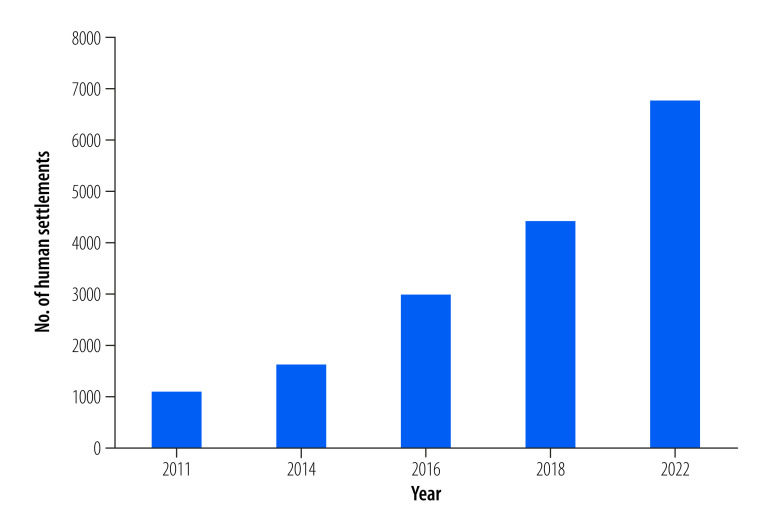
Human settlements with air quality data included in the WHO air quality database, by year of release

Since the first publication of the database in 2011, WHO has published four updates, in 2014, 2016, 2018 and 2022. Throughout the years, data coverage has improved sixfold: from 1102 human settlements (this can cover cities, towns and/or rural areas)^19^ included in 2011, to 6743 in 2022 (Fig. 1). While data on particulate matter in 2011 were available for 91 countries, the latest version of the database includes information from 118 countries (Table 1). Moreover, whereas 52% (573/1102) of settlements reported PM_2.5_ in 2011, this proportion reached 64% (3872/6050) in 2022, thus increasing the availability of relevant data for health risk and impact assessment. In the most recent database, WHO also included data on nitrogen dioxide (NO_2_), with 5667 settlements in more than 80 countries reporting NO_2_ concentrations. This expansion recognizes the importance of NO_2_ as an important health risk in cities, as well as the differences in NO_2_ distribution patterns compared with particulate matter, requiring customized policy interventions for effective air pollution reduction.

WHO: World Health Organization.

Source: WHO, 2022.^6^

**Table 1:** Countries added to the WHO air quality database since 2011

WHO region	No. of new countries included	Country^a^
Africa	4	Cameroon, Ethiopia, Kenya, Uganda
Americas	5	Bahamas, Cuba, Honduras, Paraguay, Trinidad and Tobago
South-East Asia	1	Maldives
Europe	10	Albania, Andorra, Georgia, Kazakhstan, Kyrgyzstan, Montenegro, Tajikistan, Turkmenistan, Ukraine, Uzbekistan
Eastern Mediterranean	6	Afghanistan, Bahrain, Iraq, Jordan, Morocco, Qatar
Western Pacific	3	Fiji, Lao People's Democratic Republic, Viet Nam
Non-WHO Member State	1	Liechtenstein

WHO: World Health Organization.

^a^ San Marino and Botswana were included in the 2011 air quality database but not in the 2022 version.

One of the early objectives of the WHO air quality database was to feed into efforts to derive national population exposures based on global air quality models. These models estimate air pollution levels as a spatial continuum (with resolution of about 10 × 10 km), covering the whole world.^20^^,^^21^ The use of information on pollutant emissions and their atmospheric transport and reactions, coupled with remote measurements of pollutant concentrations from satellites and calibrated with ground measurements by complex statistical models, has resulted in estimates that are well correlated with the available observations.^18^ More importantly, this approach also allows estimation of pollutant concentrations in places that lack air quality monitoring data, and encourages improvement in global availability of monitoring data. Recent methodological updates incorporate satellite data with PM_2.5_ component fractions which can, for example, enhance our understanding of source contributions and guide national policies and source-specific interventions.

The publication of the database has attracted considerable media attention – and even controversy – in many different countries, resulting in an increased awareness of the negative effects of air pollution on human health. The database also provides WHO with an opportunity to engage actively with countries on air pollution, bringing health to the centre of the discussion on policies on air pollution abatement.^22^^,^^23^

While current efforts in collecting air pollution data focus on all areas (for example, cities, towns and rural areas), initial efforts mostly concentrated on urban settings.^6^ This focus also helped to start the WHO-led BreatheLife campaign, now joined by a network of 79 cities, countries and regions, which aims to bring together actors – such as, agencies in charge of air quality management, local government and civil society groups – to share knowledge and best practices in both monitoring air pollution and curbing its levels.^24^

**Fig. 2:**
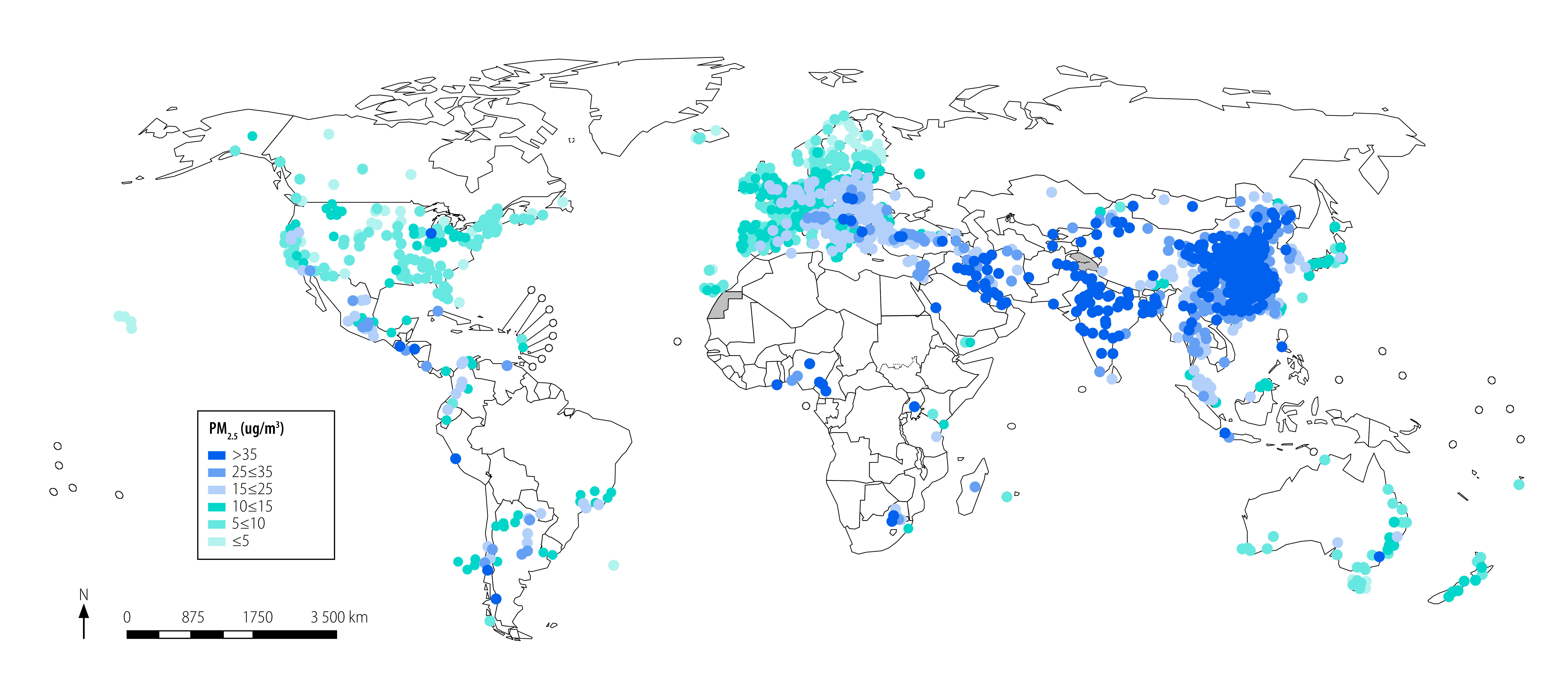
Locations of settlements with data on PM_2.5_, by number of ground measurements, 2010–2019

The coverage of ground measurements of PM_2.5_ compiled in the WHO database is not homogeneous around the world, and is concentrated in high- and middle-income countries and areas mainly in China, Europe, India and North America (Fig. 2). In the 2022 version of the database, 58% (3911/6743) of settlements are located in high-income countries; 34% (2279/6743) in upper-middle-income countries; 7.7% (519/6743) in lower-middle-income countries; and only 0.3% (20/6743) in low-income countries. The pattern is similar for NO_2,_ with more ground monitors in high- and middle-income countries.^6^ Noteworthy is the fact that in densely populated areas where measurements are still sparse, the uncertainty in the population exposure estimates is much greater and so is the calculation of the burden of disease attributable to air pollution.^18^^,^^20^ Overall, the available data show that air pollution levels are high; with PM_2.5_ and PM_10_ concentrations in 94% (6338/6743) of the settlements not complying with WHO air quality guidelines.

PM_2.5_: particulate matter with a diameter ≤ 2.5 µm.

Note: Some countries measure only or mainly PM_10_ (particulate matter with a diameter ≤ 10 µm) and are hence not reflected in this figure.

Source: World Health Organization, 2022.^6^

## Challenges

Since the first efforts to compile an air quality database, the primary and preferred sources of data have been official national and subnational reports and websites. When official data have not been available, values from research networks and/or peer-reviewed journals have been used in 13 countries.

Retrieving official data can be challenging when the responsibility of air pollution monitoring is held at the subnational level or is distributed between various national actors, making the data retrieval process less effective and more time-consuming. For example, data collection in some countries is not centralized but is rather the responsibility of cities, districts or states (for example, Australia, Brazil and Chile), and is conducted without national oversight and coordinated reporting. Another challenge is the collection of routine ambient air pollution measurements, which is the responsibility of multiple ministries such as the environment ministry, national statistical office and health ministry (for example, Qatar).

An additional challenge in compiling the WHO air quality database is the diversity of methods used to monitor and report air quality. Reasons for this heterogeneity include: the cost associated with procuring and maintaining reference grade monitors;^25^^,^^26^ lack of or differences in regulations and technical requirements to determine the acceptability of measurements; and insufficient training and maintenance of qualified technical personnel. Given the pressure to report the status of air quality, for some countries, the use of low-cost sensors is a more attractive option. These sensors have an upfront cost of a few thousand United States dollars while reference grade monitors can have a capital cost of tens of thousands of United States dollars. However, the operating cost for maintenance, software management, analysis, quality control and quality assurance of the data from low-cost sensors is not negligible and should be carefully considered, as well as data ownership.^25^^,^^27^

As regards the database, its objective is to compile existing and publicly available annual mean concentrations of PM_2.5_, PM_10_ and NO_2_ from officially operated monitoring networks. However, the data collected in the database are not homogeneous as each country may use different instruments, measurement techniques, station siting protocols and classifications. They may also apply different data reporting and aggregation methods, time coverage, and standards for data quality control and validation. Data may be missed because of language, or may not comply with inclusion criteria due to missing metadata. Because of these limitations, any direct comparisons of particulate matter and NO_2_ concentrations across countries and years must be done with caution.^6^^,^^28^

## Importance of air quality data

The increase in data availability and the collection of historical data on air pollution is important to better understand the status of air quality and monitor trends, including on emerging sources of air pollution such as wildfires and sand and dust storms, and progress in policies to reduce pollution. For example, elevated PM_2.5_ concentrations in Australia in 2019 relative to previous years highlights the impact of extensive wildfires on air quality. On the other hand, decreases in PM_2.5_ concentrations in China after 2013 illustrate the effect of national programmes to reduce air pollution.

The regular compilation and publishing of global air pollution data can also reduce restrictions on data sharing, and facilitate access to data collected by monitoring networks, by demonstrating the usefulness of a comprehensive assessment of air quality based on international data. The availability of air pollution data can stimulate health impact analysis and policy tracking activities that would otherwise be difficult.^29^ Access to these data can also support research in the field of exposure modelling and epidemiology. Although the data are not updated annually, during each update (every 2 to 3 years), capacity-building that aims to enhance the data collection process is undertaken through the training of government officials of WHO Member States via country consultation, discussions and webinars with exposure scientists (for example, atmospheric and modelling scientists) and epidemiologists. Capacity-building initiatives, such as local health impact assessment projects, are also shared by WHO Member States to promote knowledge translation.

Countries are sensitive about their air quality data, and it has taken time to build trust when monitoring the SDG indicators 11.6.2 and 3.9.1, especially to obtain input data. Yet, over the years, countries have engaged with WHO to share their data. Data ownership and understanding of the science underpinning the health impacts of air pollution are key, and supporting national institutions in monitoring air quality and conducting health impact assessments should focus on using their local data. This effort should be coupled with appropriate and sustainable capacity-building and resources. A good example of a science policy framework is the *Convention on long range transboundary air pollution*. The cooperative programme for monitoring and evaluation of the long-range transmission of air pollutants in Europe has shown the importance and value of using data validated by countries in a clearly defined and internationally established framework and methods.^30^

Over the past few years, methods to assess air quality have improved (for example, extensive use of satellites, low-cost sensors and modelling), allowing better coverage and space–time resolution of data.^18^^,^^27^^,^^31^^,^^32^ While countries may be tempted to change to these alternatives instead of maintaining reference grade monitors which are the gold standard, albeit costly, these new methods (whether measured or modelled) have not all been properly validated, and the cost–benefits of the individual and combined methods need to be assessed. As well as measuring air quality, it is important that countries share the data with the public in a way that is easy to access and interpret. While air quality indices are informative for risk communication, they are limited in comparability and harmonization; publishing air pollutant values is therefore preferable, as these data are more informative and allow direct comparison.

## Going forward

A unique opportunity has arisen to encourage and strengthen health-sector engagement in air quality, as countries are considering revising their national ambient air quality standards to better align with the 2021 update of the WHO air quality guidelines to protect public health. Health considerations need to be better integrated in policies of those sectors that are substantial emitters of air pollutants. Currently, only 64% (124/195) of countries have legal requirements to monitor air quality, and 9% (18/195) of them integrate WHO’s recommendations in their national ambient air quality standards.^33^^–^^35^ It is important to recognize that the WHO air quality guidelines do not specify end goals that define success or failure, but rather they offer guidance of what better health can look like with cleaner air. In a perfect world, there would be no air pollution, but in the absence of that ideal, policy-makers must acknowledge that any action they take to reduce air pollution is a step in the right direction for public health.

Advances in exposure assessments – for example, low- and middle-income countries providing exposure–response functions for WHO’s 2021 air quality guidelines – reflect methodological improvements and progress in the scientific assessment of the health effects of air pollution. Local data compiled for regulatory purposes have been, and continue to be, regularly used to assess population exposure to ambient air pollution; monitor the effectiveness of policies and actions; assess health impact; derive burden of disease estimates; and undertake epidemiological research.^29^ Yet, data availability and quality are often not ideal, and fail to capture human exposure and measure exposure inequalities. For example, the number of monitors available to map population exposure tends to vary greatly across the world: 2–3 monitors per million persons in Europe; 0.5 monitors per million persons in Japan.^36^ In Africa, data are available from 41 cities in 11 of 47 countries, which means that no data monitoring exists for tens of millions of people.

Despite increased advocacy, the increase in monitoring of air quality has not been equal across the regions. The health community therefore needs to increase engagement in monitoring air quality to facilitate health-risk assessments, especially in view of the growing availability of new methods and technologies, for example, low-cost sensors and modelling or a combination. While low-cost sensors cannot replace reference grade monitors for long-term health impact assessments, several platforms have combined data from low-cost sensors with data from official reference grade monitors to provide global overviews of real-time air pollution that can raise awareness and inform public action.^37^^,^^38^

To strengthen engagement of the health sector to support national health impact assessments, WHO established the global air pollution and health technical advisory group to advise on methodological improvements for measuring air pollution exposure and its health effects.^39^ The group has provided guidance on several reports that have documented advances in methods to assess exposure to air pollution,^40^ and epidemiological studies,^41^ which can help countries increase local monitoring and health impact assessments. In the near future, the advisory group will support actions to respond to emerging threats, such as wildfires, and sand and dust storms, and guide the development of a global database on air pollution policies.

Currently, WHO is collaborating with academic institutions, and governmental and nongovernmental agencies across the world to develop training modules that cover exposure and health impact assessments among others topics, which can be conducted in person or via the OpenWHO platform.^42^ These training modules will also be adapted into webinars, which will help build the capacity of the health workforce to engage in multisectoral action with the transport and energy sectors to ensure health is considered in policy planning.^43^

WHO has been working closely with the World Meteorological Organization and UNEP over the years on air quality issues at the global level, both on advocacy and global modelling.^23^^,^^24^ WHO and UNEP have a longstanding collaboration at the regional level in ministerial processes in Asia and Latin America, which has ensured harmonization of the communication strategy, and resolutions endorsed by ministries of the environment that support WHO’s work on air quality and health. In Europe, WHO collaborates with the United Nations Convention on Long-Range Transboundary Air Pollution as the secretariat of its joint task force on health.^44^ This collaboration was further strengthened by the UN inter-agency working group on reporting on SDG 11.6.2, and through the compilation of tools and guidance documents covering standards, measurements, modelling, and health and economic impact assessments, among others, that can support countries in working towards the WHO air quality guidelines.^45^

Collective harmonization is needed of national ambient air quality standards, definitions for monitoring air quality, and standardized methods and techniques, so that data collection and monitoring of progress can be efficiently conducted and can be used to inform decision-makers on what is working best to protect public health from air pollution.

To conclude, with almost 50 years of experience compiling air quality data, WHO’s air quality database is important to demonstrate the magnitude and global distribution of health risks due to air pollution. The database can also help Member States to identify tools that can enhance their capacity to assess and reduce air pollution health risks, and actions to empower multiple sectors to advocate for clean air for healthier populations.
