# Corrigendum

**DOI:** 10.2471/BLT.24.100124

**Published:** 2024-01-01

In: Shairsingh K, Ruggeri G, Krzyzanowski M, Mudu P, Malkawi M, Castillo J, et al. WHO air quality database: relevance, history and future developments. Bull World Health Organ. 2023 Dec 1;101(12):800–807, 

on page 804, the funding section should read as follows:

